# Development of an Antioxidant-Rich Sugar-Free Plantain Candy and Assessment of Its Shelf Life in a Flexible Laminate

**DOI:** 10.17113/ftb.62.02.24.8141

**Published:** 2024-06

**Authors:** Poulami Sarkar, Paramita Bhattacharjee, Bidhan Das

**Affiliations:** 1 Department of Food Technology and Biochemical Engineering, Jadavpur University, 188, Raja S.C. Mallick Road, 700032 Kolkata, West Bengal, India; 2 Eastern Regional Centre, Indian Institute of Packaging, Block C. P. 10, Sector V, Salt Lake, Bidhan Nagar, 700091 Kolkata, West Bengal, India

**Keywords:** minimal additives, biomolecules, sensory properties, physicochemical and biochemical characteristics, flexible packaging, shelf life

## Abstract

**Research background:**

Candy is a popular confection worldwide, and it would be beneficial to society if it were converted into a source of antioxidant molecules to eliminate its adverse health effects. The amount of antioxidants available even in fruit candies is questionable due to the high thermal processing losses they undergo and the presence of various food additives. Plantains (*Musa paradisiaca*) are less known as good sources of biotherapeutic antioxidants, namely l-tryptophan, serotonin and melatonin, and consumption of this highly nutritious fruit is limited to underdeveloped and developing countries. The objectives of this study are: to develop a functional antioxidant-rich sugar-free plantain-based candy with valuable contents of the mentioned biomolecules in synergy; and to ensure its extended shelf life without compromising its physicochemical properties and functionality by wrapping it with a suitable packaging laminate.

**Experimental approach:**

To accomplish the first objective, lyophilized plantain powder, sorbitol and mannitol were used as base materials with minimal additives under minimal processing conditions to reduce processing loss. Sensory, proximate, physicochemical and phytochemical properties, including the antioxidant synergy among the mentioned biomolecules of the developed candies were evaluated. For the second objective, the candies were enclosed in two different flexible packaging laminates and the optimal packaging was determined based on the microbiological safety and sensory appeal of the wrapped candies. Subsequently, the above-mentioned properties of the packaged (in the most suitable laminate) candies were evaluated at regular time intervals during storage for assessment of their shelf life.

**Results and conclusions:**

The candy had a characteristic flavour of plantain, uniform dark brown colour, rich mouthfeel, pleasant aroma, moderately hard texture and moderate sweetness, along with high antioxidant activity and considerable content of l-tryptophan, serotonin and melatonin (present as a synergistic consortium). During storage of the packaged candy under ambient conditions, it remained microbiologically safe for up to 56 days, and also maintained sensory attributes, antioxidant activity and synergy compared to the control candy.

**Novelty and scientific contribution:**

This newly developed semi-hard sugar-free candy with high antioxidant content, containing three important antioxidants, namely l-tryptophan, serotonin and melatonin, could be a good source of biotherapeutic molecules and a substitute for commercial candies consumed globally.

## INTRODUCTION

Green plantains (*Musa paradisia*ca) are widely consumed in Asia, Africa and South America, particularly for their high content of carbohydrates (including resistant starch), dietary fibre and minerals, such as iron (*1*). However, they are less well known for their abundant antioxidants. Our previous studies have shown that green plantains are a rich source of antioxidants, especially the monoamine neurotransmitter serotonin (5–135 µg/g), the neurohormone melatonin (0.5–7.2 µg/g), and their biosynthetic precursor molecule, the essential amino acid l-tryptophan (*2*). In some less developed countries with food shortages, plantains are a staple food (*3*). The development and consumption of food products made from plantains are limited to wine, beer, pickles, chips, cookies, bread and cakes (*3*-*7*). However, knowledge about the production of candies from plantains is limited.

Candies are very popular with all age groups worldwide. In America, 26 % of the total population (aged ≥2 years) consume on average 40 g of candy (176 kcal) per day (*8*). However, the added sugars and saturated fats in candies are specifically responsible for several health-debilitating metabolic and cardiovascular disorders (*8*). In addition, the presence of synthetic antioxidants such as butylated hydroxyanisole (BHA) and butylated hydroxytoluene (BHT) (*9*) in commercially available candies has negative effects on health (*10*). Even in candies made from natural fruit or vegetable pulp and juices, the natural antioxidant content is compromised by heat degradation during thermal processing. Furthermore, the presence of various food additives in addition to the high fat and sugar content may hinder the utilization of the health-promoting antioxidants (if they do not degrade during processing). These are not compatible with the labelling of the fruit/vegetable candies rich in antioxidants. We envisage that minimal processing with minimal amount of food additives and low sugar content can result in a plantain-based candy that is truly rich in antioxidants, thereby minimising adverse health effects.

Some recent studies have documented the formulation of candies with substitutes for commonly used ingredients to circumvent the harmful effects of candy consumption. Few researchers have successfully used spices such as a mixture of turmeric, ginger, liquorice, white wormwood and citrus extracts (*11*) to develop antioxidant-rich hard candies. Several studies have reported the production of fruit candies using various fresh fruits, including unripe mango (*12*). Acetic acid, alcoholic and lactic acid-fermented apple juice products (*13*), as well as fermented algae such as *Spirulina* (*14*) have been used to produce chewy candies. The production of soft candies (gummy jellies) by adding pomegranate juice (*15*) and other natural extracts is well documented. Some researchers have also explored preparing them using starch or sugar substitutes such as inulin (*16*). The use of natural antioxidants such as rosemary extract with stevia (*17*) together with fructan fibre (chicory inulin and fructooligosaccharides), green propolis extract with fructan (*18*) and sage extract with inulin-gelatine-fructooligosaccharides (*19*) has also received recognition. However, there is a dearth of information on the production of hard and semi-hard candies.

Studies on the formulation of hard and semi-hard candies using sugar substitutes are scarce, except for a single report by Jeon *et al.* (*20*) on the formulation of a nutraceutical hard candy using isomalt, maltitol syrup and xylitol as sucrose substitutes. They used extracts from the fruit of *Cudrania tricuspidata* (manadarin melon berry), lemon and ginger as sources of natural antioxidants. However, to the best of our knowledge, there is no report on the development of sugar-free hard or semi-hard plantain candies rich in natural antioxidants that emphasizes the molecular identities of the important biotherapeutic molecules, particularly l-tryptophan, serotonin and melatonin.

Therefore, the first objective of the present study is to develop a sugar-free candy rich in antioxidants that could be a potential dietary source of l-tryptophan, serotonin and melatonin without interfering with the natural antioxidant synergy (*21*). The main obstacle in developing antioxidant-rich foods lies in the preservation of natural food synergy (*22*), which, if altered, would render the consortium of antioxidants present in the finished food product harmful rather than beneficial *in vivo* (*21*). The current study therefore focused on the modification of the candy processing parameters by (*i*) using minimal amount of food additives to allow the unimpeded release of antioxidants, (*ii*) minimising the loss of heat-sensitive molecules (especially serotonin and melatonin), (*iii*) applying minimal processing, not only to preserve the natural antioxidant synergy of green plantains, but also to avoid the destructive side effects of consuming ultra-processed food products (*23*) and (*iv*) using substitutes for the substances known as candy doctors (*9*, *24*) to provide a nutraceutical yet palatable sugar-free candy.

The second objective of the study is to evaluate the shelf life of the newly formulated designer candies after packaging in commercially available flexible laminates (mainly foil wrappers) to ensure an extended shelf life without compromising the physicochemical properties of the candies throughout the storage period.

The novelty of this research is the development of a value-added food product from a widely cultivated healthy fruit. This could be a unique confectionary product containing important biotherapeutic antioxidant molecules that promotes better use of plantains around the world.

## MATERIALS AND METHODS

Authenticated Indian (‘desi’ variety) green plantain (*Musa paradisiaca*) grown in Narendrapur (Kolkata, India) was used for the present study. The details of authentication, procurement and selection of plantains were described in detail in our previous publication (*2*). Individual plantains were carefully selected for this study based on visual assessments of their colour (only the green, unripe ones were chosen), surface morphology, texture (firm and free from blemishes, bruises and black spots) and size and mass (approx. (175±10) g on average with approx. (120±5) mm diameter).

The following food-grade chemicals were used as candy ingredients: d-sorbitol (Bioven Ingredients, Noida, India), d-mannitol (Mitushi Biopharma, Ahmedabad, India), gum acacia (Minimal Confections, Surat, India), SiO_2_ (Bioven Ingredients), sorbic acid (Bakersville India Pvt. Ltd., Indore, India) (all in powder form) and vanilla essence (Superb Formulations Pvt. Ltd., Kancheepuram, India). Standards such as l-tryptophan, serotonin and melatonin, speciality chemicals such as 2,2-diphenylpicrylhydrazyl (DPPH), high performance liquid chromatography (HPLC)-grade acetonitrile, acetic acid and water were purchased from Merck Life Science Pvt. Ltd. (Mumbai, India). Folin-Ciocalteu reagent, gallic acid and all other analytical research-grade chemicals and culture media for microbiological analyses were purchased from HiMedia (Mumbai, India). For solid-phase extraction, Quick, Easy, Cheap, Effective, Rugged and Safe (QuEChERS) kits were purchased from Agilent Technologies, Wilmington, DE, USA.

**Fig. S1:**
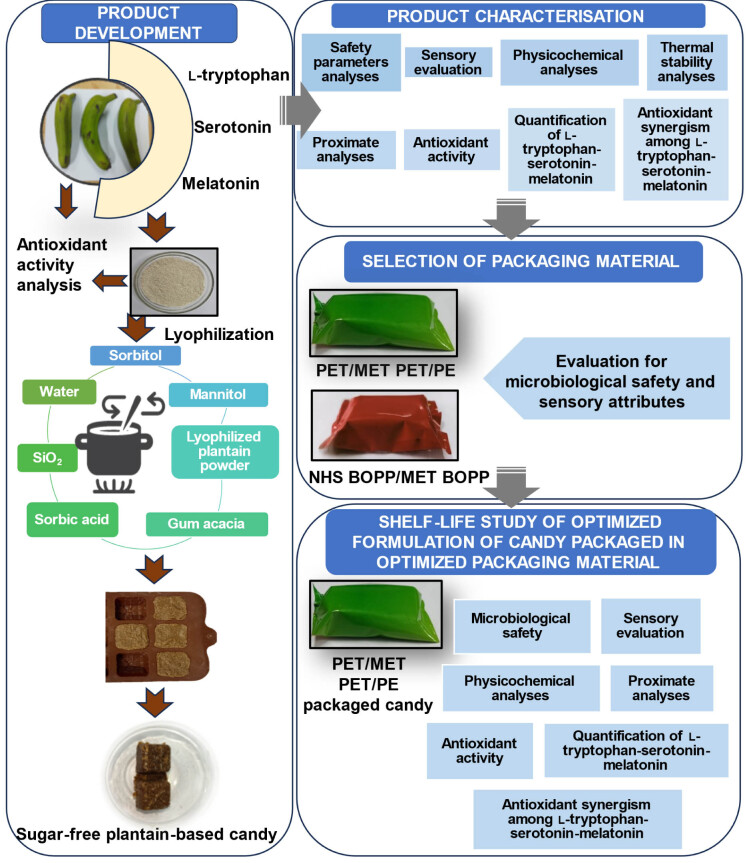
Experimental design of the present study

The experimental design of the present study is shown in Fig. S1. For the development of a new plantain-based sugar-free candy with high antioxidant content, it was first necessary to determine the antioxidant potential of raw plantain and the content of the desired molecular antioxidants, namely l-tryptophan, serotonin and melatonin. Three plantain samples were randomly selected from the procured sample lot and analysed for their antioxidant activity, especially their total phenolic content (TPC), DPPH radical scavenging activity and Fe(III) ion-reducing antioxidant power (FRAP), as well as their content of l-tryptophan, serotonin and melatonin. The details of the analytical methods can be found in an earlier publication by the authors (*2*).

### Preparation and characterisation of lyophilized plantain powder

The fully mature green plantains were thoroughly washed, then cut together with the peel into very small cube-shaped pieces (except for the stem and the black tip) and frozen in an ultra‐low temperature freezer (Premium C340; New Brunswick Scientific, Enfield, CT, USA) at −80 °C for 24 h and finally dried in a bench top freeze dryer (FDU-1200; Eyela, Tokyo, Japan) at −45 °C and 14.4 Pa to a final moisture content below 6 %. The freeze-dried plantain pieces were then ground in a mixer grinder (HL 1618; Philips India Limited, Chennai, India) to obtain a powder with a particle size of <300 µm and the same was subjected to solvent extraction according to the method described by Dal-Bó and Freire (*25*), with some modifications. The lyophilized plantain powder (0.5 g) was first dissolved in dimethyl sulfoxide (DMSO) (in a 1:10 solid/solvent ratio) in an incubator shaker (IS 02; Incon Instruments Company, New Delhi, India) at 1×*g* and 30 °C for 2 h, after which the mixture was centrifuged (R-8C; Remi, Mumbai, India) at 1000×*g* for 15 min. The supernatant was analysed for its antioxidant activity (in terms of TPC, DPPH radical scavenging activity and FRAP value) in accordance with the procedures followed for the raw plantains (*2*). The lyophilized plantain powder was then used in the preparation of the candy.

### Formulation of a plantain-based, sugar-free candy rich in antioxidants

The ingredients selected for the formulation of the antioxidant-rich candy, their functions and the safe limits of use (*26*) are given in Table S1 (*26*, *27*). Several preliminary tests were conducted to determine the exact amount of each ingredient for the candy formulation. Sugar alcohols such as d-sorbitol and d-mannitol were selected as base ingredients (*27*) for the candy formulation, mainly because they provide fewer calories (3 and 2 cal/g, respectively) (*28*), have negligible glycaemic indices (4 and 2, respectively) (*29*) compared to regular table sugar (4 cal/g; glycaemic index 65) and also serve as plasticizers (*9*). Since d-sorbitol and d-mannitol have a relative sweetness of 0.6 and 0.5–0.6, respectively (*30*), compared to sucrose (relative sweetness 1), it was assumed that the sweetness of the formulated candy would not be affected. Gum acacia as a thickening agent was varied in the mass fraction range of 3-35 % based on the total mass of the candy ingredients. The preliminary tests showed that a mass fraction of 30 % (based on the total mass of the candy ingredients) could give the candy the desired ‘stand-up’ property (*24*). The amount of lyophilized plantain powder was varied in a mass fraction range of 55-85 % of the total mass of the candy ingredients. It was found that a mass fraction of 65 % conferred desirable sensory properties to the candy. Mass fractions less or more than 65 % resulted in unsuitable texture of the candies, *i.e*. they were either too hard or poorly formed, *i.e*. they lacked structural integrity (data not shown).

To formulate a plantain-based candy sample, sorbitol, mannitol, gum acacia, SiO_2_, sorbic acid and water were mixed with a spatula and the mixture was heated (180-210 °C) with constant stirring (to prevent lump formation) until it obtained the desired caramel colour. The lyophilized plantain powder and vanilla essence were added to the mixture after it had cooled to 60 °C to preserve the desired heat-labile antioxidants. Then sorbitol and mannitol powder were added to the mixture and allowed to set in candy moulds for 75 min. In this process, the use of food additives was kept to a minimum. No synthetic food colours, organic acids (acidulants) or synthetic antioxidants were used in the formulation of the candies, unlike those used in the production of commercial candies (*9*). In the formulation of the experimental control candies (without plantain), all ingredients and procedures were similar to those used for formulating plantain-based candy, with the exception that no plantain powder was used. The mass and dimensions of the resulting control and plantain-based candy samples were measured using a balance (BSA 224S-CW; Sartorius AG, Göttingen, Germany) and a vernier calliper (532 series; Mitutoyo, Kawasaki, Japan), respectively.

### Assessment of the safety parameters of control sample and plantain-based candies

The analyses described below were performed to evaluate the safety profile of the newly formulated candies.

#### Microbiological assessment

The microbial count of the bacteria and fungi (expressed as CFU/g) in candies was evaluated based on total plate counts of the formulated candies using the pour plate method (*31*).

#### Energy dispersive X-ray analyses

To ensure the absence of toxic heavy metals such as Pb, Ti, Hg, Ni, As, Si and Mo, which may be present in the raw material (plantains) or may have been acquired during the production of the candies, energy dispersive X-ray (EDX) analyses of control and plantain-based candies were performed using scanning electron microscope INSPECT F50 (FEI Company, Hillsboro, OR, USA).

### Sensory and physicochemical characterisation of control sample and plantain-based candies

The analyses described below were conducted to evaluate the eating quality of the newly formulated candy samples, primarily with regard to the characteristic plantain flavour (by sensory evaluation) and mouthfeel (by both sensory and textural evaluation).

#### Sensory evaluation

The sensory evaluation of the control sample and plantain-based candies was carried out using an acceptability test based on a 9-point hedonic scale (where 9 stands for ‘like extremely’ and 1 for ‘dislike extremely’). The candy samples were served to the participants using the serving protocols for sensory evaluation of food and beverages described in ASTM E1871-17 guide (*32*). Fifteen men and fifteen women (35–45 years old) were selected from faculty members and research scientists of our university to form a semi-trained sensory panel (*33*). The panellists were selected based on their interest and their performance was evaluated using screening tests with the control sample. Prior to sample evaluation, they were familiarised with the sensory attributes of the prepared candies and the commercially available hard candies (Mango bite and Poppins) and asked to evaluate the experimental candies in terms of appearance, texture, colour, taste, flavour, mouthfeel, aftertaste and overall quality. The sensory evaluation was conducted in the morning from 10 am to 12 pm in a well-ventilated room with white light. Unsalted crackers and water (to rinse the palate) and expectoration cups (covered) were provided to all participants before each evaluation if they did not wish to swallow the samples (*34*). Three specimens of each individual sample were served in each session and the mean scores (rounded) were presented graphically in radar plots.

#### Colour and texture analyses

Instrumental analyses of the colour and texture properties of the newly formulated candy samples were performed to validate the sensory ratings provided by the human judges (as described above) and to remove subjectivity. CIE colour value analysis (L* for lightness, a* for red-green and b* for yellow-blue) of control and plantain-based samples was conducted using a colour reader (CR-10 Plus, Konica Minolta Inc., Osaka, Japan) (*2*). The texture profiles of control sample and plantain-based candies were analysed by the two-bite compression test using a texture analyzer (TA.XT Express, Stable Micro Systems, Godalming, UK) in accordance with the method described by Sarkar *et al.* (*2*) and the data were processed using Exponent Lite Express software v. 6.1.4.0 (*35*). A cylindrical aluminium probe (P/5) of 5 mm in diameter was used for the test, and the sample was oriented so that the compression was symmetrical to its geometric centre.

### Microstructure analyses of control samples and plantain-based candies

The following analyses were carried out for the detailed structural characterisation of the newly formulated candies.

#### Field emission scanning electron microscopy

The surface morphology of the new designer candies was analysed using field emission scanning electron microscopy (FE-SEM; INSPECT F50; FEI Company) at an operating voltage of 5 kV. The samples were first dried under vacuum (6–7 Pa) and then coated with gold using a coating device (Q150R ES; Quorum Technologies Ltd., Ashford, UK).

#### X-ray diffraction analysis

The X-ray diffraction (XRD) patterns of the control samples and the plantain-coated candies were analysed at room temperature ((23±2) °C) using a diffractometer (D8 Advance; Bruker, Billerica, MA, USA) and Cu-Kα1 radiation at a wavelength of 0.15406 nm. Measurements were performed at a voltage of 40 kV/40 mA. The XRD data were collected to cover an angular range (2 *θ*) of 30–100 ° at a width of 0.01 ° and a counting time of 0.5 s/step.

### Thermal stability assessment of control sample and plantain-based candies

It is necessary to study the thermal stability of the candies in order to evaluate the thermal (and thus structural) changes that the candies undergo during storage and transport after packaging under ambient conditions.

#### Thermogravimetric analysis

To predict the thermal stability of the control samples and the plantain-based candies, a thermogravimetric analysis (TGA) was performed using the TGA 4000 (Perkin Elmer, Hopkinton, MA, USA). In this analysis, an empty platinum crucible containing α-alumina powder was used as a reference. The samples, which were placed individually in a hermetically sealed aluminium pan, were heated from 51 to 600 °C and scanned at a rate of 10 °C/min under a nitrogen flow of 20 mL/min.

#### Differential scanning calorimetry analysis

For differential scanning calorimetry (DSC) analysis, the control sample and the plantain-based candies were equilibrated at -80 °C for 5 min, then heated to 130 °C at a rate of 5 °C/min and held at this temperature for 1 min. The samples were then cooled to −50 °C at a rate of 5 °C/min and held at this temperature for 5 min. The samples were then reheated to 110 °C at a rate of 5 °C/min. The heat-cool-heat method was used, which is considered suitable for hard candies (*36*). For this study, the samples were analysed using a DSC Q2000 (TA Instruments, New Castle, DE, USA).

### Analysis of the water activity of control sample and plantain-based candies

The water activity (*a*_w_) of the control sample and the plantain-based candies was determined according to AOAC method 978.18-1978 (*37*) to assess their shelf stability.

### Proximate analysis of control sample and plantain-based candies

Proximate analyses of the control sample and the plantain-based candies were conducted in accordance with the standard methods, including estimation of the percentages of moisture (*38*), protein (*39*), crude fat (*40*), ash (*41*), crude fibre (*42*) and total carbohydrates by difference, all on a dry mass basis (dm) (*2*).

### Acidity and solid content of control sample and plantain-based candies

To determine acidity and solid content of the control sample and the plantain-based candies, they were dissolved in DMSO following the protocol described for the lyophilized plantain powder (described earlier). The resulting supernatants were stored at −20 °C prior to analyses. These were subjected to analyses of total titratable acidity (TTA) as a percentage of malic acid equivalents (*33*) using standard NaOH (0.1 M), pH (Cyberscan PC510m pH metre; Eutech Instruments Pvt. Ltd., Singapore City, Singapore) (*2*), total insoluble solids (gravimetric) and total soluble solids as °Brix (*21*) (OptiDuo refractometer; Bellingham + Stanley Ltd., Tunbridge Wells, UK).

### Antioxidant properties

As the control sample consisted mainly of sugar alcohols and contained no additional source of natural antioxidants, the evaluation of antioxidant properties was only performed for the plantain-based candies.

#### Assessment of the antioxidant activity of plantain-based candies

The supernatant obtained from processing of the plantain-based candies was also used for the determination of reducing power on dry mass basis expressed in μg of butylated hydroxytoluene (BHT) equivalents per g (*43*), TPC expressed in mg of gallic acid equivalents (GAE) per 100 g using the Folin-Ciocalteu method (*44*), DPPH radical scavenging activity expressed as IC_50_ values (mg/mL), and FRAP value expressed as FeSO_4_ in mM/100 g (*2*) using standard spectrophotometric methods and UV-Vis double beam spectrophotometer (Halo DB-20; Dynamica Scientific Ltd., Newport Pagnell, UK).

#### Determination of l-tryptophan, serotonin and melatonin by HPLC-photo diode array (PDA) analysis using QuEChERS-solid phase extraction (SPE) extract of plantain-based candies

For the extraction of l-tryptophan, serotonin and melatonin from the plantain-based candies, the QuEChERS-SPE method was followed according to the procedure described in our previous publication (*2*). Candies (15 g) were crushed using a mixer grinder (HL 1618; Philips India Limited) and placed in a clean centrifuge tube, to which 1 % acetic acid in acetonitrile solution and the contents (MgSO_4_ and NaCl) of an SPE AOAC packet were added. After thorough mixing using a vortex (iSwix VT; Neuation Technologies Pvt. Ltd, Gujarat, India), the tube was centrifuged at 1500×*g* for 1 min in a centrifuge (R-8C; Remi, Mumbai, India). A volume of 1 mL of supernatant was withdrawn and added to the dispersive SPE (dSPE) sample cleanup tube (containing primary and secondary amine, C18 sorbent (trifunctionally bonded C18 silica), graphitized carbon black and MgSO_4_) and thoroughly mixed. The dSPE sample cleanup tubes were again centrifuged at 1207×*g* for 5 min using a microspin centrifuge (TC-4815D; Eltek, Haryana, India). The supernatant, *i.e.* the extracted sample was filtered using a micro syringe filter (0.22 µm nylon) and stored in an amber-coloured glass vial at −20 °C for further analyses. HPLC-PDA detection of l-tryptophan, serotonin and melatonin was conducted in accordance with the procedure developed by our laboratory (*45*) using a C18 reversed phase HPLC system (LC-Net-2/ADC, PU-4180HPLC pump, DG-4000-04 degasser and MD-4015 detector; Jasco, Tokyo, Japan). HPLC-grade methanol and 1 % HPLC-grade acetic acid in HPLC-grade water were used as mobile phase solvents in gradient mode, each at a flow rate of 1 mL/min. A PDA detector with a deuterium (D2) lamp set at 280 nm was used for continuous monitoring of the eluents. The peaks of l-tryptophan, serotonin and melatonin were identified based on the retention time of their corresponding sigma standards.

#### Assessment of antioxidant synergism among l-tryptophan, serotonin and melatonin in plantain-based candies

All naturally occurring antioxidants in a food are always present in a synergistic consortium (*22*). Once isolated, antioxidants can act either synergistically or antagonistically (*46*). Therefore, it is of utmost importance to achieve antioxidant harmony without disrupting the natural synergy of foods if the benefits of antioxidants are to be realised *in vivo*. Preservation of the naturally occurring synergism among the three antioxidants mentioned in plantain-based candies was one of the main objectives of this study to ensure that the designer candy is an antioxidant-rich product. To evaluate the ‘food synergy’ in the developed candy, the synergism among l-tryptophan, serotonin and melatonin was assessed *in vitro* by determining the synergistic effect using the DPPH radical scavenging ability of pure chemical standards of l-tryptophan, serotonin and melatonin separately at different concentrations similar to those in plantain-based candies and a mixture containing the above antioxidants at the same concentrations (as determined by HPLC-PDA analysis of plantain-based candies). The percentage of experimental and theoretical scavenging capacity and synergistic effect were calculated in accordance with the method described by Chakraborty and Bhattacharjee (*21*). A synergistic effect greater than 1 indicates the maintenance of natural synergism amongst these three biomolecules.

### Packaging and shelf life study of wrapped candies

In order to market the newly developed candy, it is important to know its storage properties under packaged conditions. For this purpose, two flexible packaging laminates, commonly used for commercial candy packaging, provided by the Indian Institute of Packaging, Kolkata, India, were tested for the study. The flexible 2- and 3-ply packaging laminates in the form of sheets with uniform dimensions consisted of non-heat-sealable biaxially oriented polypropylene *δ*(NHS BOPP)=12 µm, white pigmented extrusion-coated polyethylene *δ*=8 µm and metallized biaxially oriented polypropylene *δ*(MET BOPP)=15 µm laminate labelled NHS BOPP/MET BOPP, and polyethylene terephthalate *δ*(PET)=12 µm, white pigmented extrusion coated polyethylene *δ*=8 µm, metallized polyethylene terephthalate *δ*(MET PET)=12 µm and polyethylene *δ*(PE)=20 µm laminate labelled PET/MET PET/PE. Their characteristic chemical and mechanical properties (*i.e*. thickness, area density in g/m^2^ (grammage), water vapour transmission rate (WVTR), oxygen transmission rate (OTR), and tensile strength) were evaluated using standard methods (*47*) and are shown in Table 1.

**Table 1:** Mechanical and chemical properties of different packaging laminates

Test	PET/MET PET/PE	NHS BOPP/MET BOPP
*δ*​/µm​	(52.0±0.5)^a^	(40.0±1.0)^b^
(*m*/*A*^2^)/(g/m^2^​)	(59.0±0.4)^a^	(35.0±0.8)^b^
WVTR/(g/(m^2^∙day)) Temperature=(38±1) °C RH=(90±2) %	(0.13±0.05)^a^	(0.12±0.08)^a^
OTR/(cm^3^/(m^2^∙day at 0.101 MPa) Temperature=25 °C)	(1.08±0.05)^a^	(56.7±1.6)^b^
Tensile strength/(kg/cm^2^)	Machine direction: (1028±2)^a^	Machine direction: (725±2)^b^
	Transverse direction: (875±2)^a^	Transverse direction: (645±1)^b^

Data are mean value±S.D. of three samples. Different letters in a row indicate significant differences at p<0.05 level. PET/MET PET/PE=polyethylene terephthalate (*δ*=12 μm), white pigmented extrusion coated polyethylene (δ=8 µm), metallized polyethylene terephthalate (*δ*=12 μm) and polyethylene (*δ*=20 μm), NHS BOPP/MET BOPP=non-heat-sealable biaxially oriented polypropylene (*δ*=12 μm), white pigmented extrusion-coated polyethylene (*δ*=8 µm) and metallized biaxially orientated polypropylene (*δ*=15 μm), WVTR=water vapour transmission rate, OTR=oxygen transmission rate, RH=relative humidity, *δ*=thickness, *m*/*A*^2^=grammage

#### Selection of the best packaging laminate (wrapping material) based on the microbiological safety of the packaged candy

To select the best flexible packaging laminate, the microbial load and the sensory properties of the candy samples (control sample and plantain-based candies) packaged in the said laminates were evaluated over a period of three months. Freshly prepared batches, each consisting of 72 control samples and 72 plantain-based candy samples (the batch size was determined based on the sample quantity required for the regular physicochemical analysis), were immediately packaged in foil wrappers of NHS BOPP/MET BOPP laminate (36 control samples and 36 plantain-based candies) and PET/MET PET/PE laminate (36 control samples and 36 plantain-based candies), leaving negligible void spaces inside the packages. The package was then sealed with a heat sealer (Delta Seal V2; Sevana Traders and Services Pvt. Ltd., Cochin, India). The packaged candies were stored under ambient conditions ((27±2) °C and (80±2) % RH). Three randomly selected samples were subjected to microbial and sensory evaluation on the first day of each week using the methods described above.

#### Shelf life assessment of candy packaged in the selected wrapper

The best packaging laminate for the newly designed candy was selected in terms of microbial safety and organoleptic acceptability of the packaged candies. A comprehensive shelf life study was then carried out with a control sample and plantain-based candies. Each batch consisted of 40 candy samples (the reasons for the choice of the said batch size were explained above) wrapped in the best packaging laminate. Changes in microbial load, sensory attributes and physicochemical properties (moisture content, alterations in CIE colour values, texture profile analysis (TPA)), antioxidant efficacy (TPC, DPPH radical scavenging activity, FRAP values), l-tryptophan, serotonin and melatonin content and antioxidant synergy were assessed throughout the storage period according to the methods described previously. Each week, three candies were randomly sampled for analyses at regular intervals. For candies approaching the end of their shelf life, microbiological analysis was performed on all days of storage.

### Statistical analysis

All data shown are the mean value±S.D. of the data obtained from three candy samples. Student’s *t*-test to evaluate the individual and interactive effects of two variables and Duncan’s multiple range test to determine significant differences among mean values were carried out using IBM SPSS Statistics software v. 26 (*48*). A value of p≤0.05 was considered statistically significant to detect differences in all tests.

## RESULTS AND DISCUSSION

### Antioxidant properties of raw plantain and its lyophilized powder

The plantains obtained for this study had a relatively high total phenolic content (TPC) expressed as gallic acid equivalents (GAE) on dry mass (dm) basis 573.17 mg/100 g, DPPH radical scavenging activity expressed as IC_50_ value 5.59 mg/mL, FRAP value expressed as FeSO_4_ 3438.12 mM/100 g dm and l-tryptophan, serotonin and melatonin mass fractions 102.45, 4.56 and 2.08 µg/g, respectively. The lyophilized plantain powder also exhibited remarkable TPC expressed as GAE 865.48 mg/100 g dm, DPPH radical scavenging activity as IC_50_ value 0.56 mg/mL and FRAP value as FeSO_4_ 5528.95 mM/100 g dm, an increase of 50.99, 89.96 and 60.81 %, respectively, compared to the raw plantains, possibly due to the concentration effect. A similar increase in antioxidant activity was also reported by Dal-Bó and Freire (*25*) in lyophilized avocado pulp powder compared to fresh avocado pulp. Due to the increased antioxidant activity, homogeneity and low moisture content (<6 %) of the lyophilized plantain powder compared to fresh plantain, the powder was used instead of fresh plantain pulp in the development of the designer candy to obtain an acceptable texture and low water activity in the candy (determined by preliminary trials).

### Mass and dimensions of control sample and plantain-based candies

The mass of the control candies was (20±2) g, while the mass of the plantain-based candies developed from the above-mentioned plantains was (24±2) g. The dimensions of both types of candy were as follows: *l*=4 cm, *b*=2 cm and *h*=2 cm. From 100 g of raw green plantains, 4 candies with the above dimensions were produced (Fig. S1).

### Safety aspects of control sample and plantain-based candies

Safety is of paramount importance for all consumable food products. Any microbial or heavy metal contamination that exceeds the safe limits can lead from mild to severe health problems and can even be fatal for the consumer. The total bacterial and yeast/mould count (no growth was detected in both samples on the first day) of the control sample and the plantain-based candies was within the safe consumption limits as per the FSSAI guidelines (*49*), which state that the bacterial and fungal count in thermally processed (except by pasteurisation, which is carried out at a temperature of less than 100 °C) fruit and vegetable products should not exceed 1000 and 100 CFU/g of food, respectively.

**Fig. 1:**
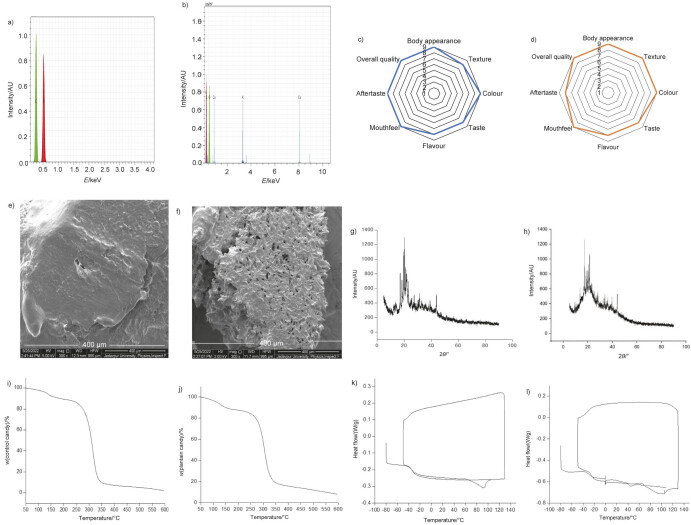
Physicochemical properties of control candy sample and plantain candy sample after formulation. The energy dispersive X-ray spectra of: a) control candy sample and b) plantain candy sample. Radar plots of hedonic scores obtained by sensory analyses of: c) control candy sample and d) plantain candy sample, where each value represents mean±S.D., *N*=3. Field emission scanning electron microscopy images of: e) control candy sample and f) plantain candy sample. X-ray diffraction spectra of: g) control candy sample and h) plantain candy sample. Thermogravimetric graphs of: i) control candy sample and j) plantain candy sample. Differential scanning calorimetry thermographs of: k) control candy sample and l) plantain candy sample

The EDX analyses of the control sample and the plantain-based candies (Fig. 1a and Fig. 1b, respectively) showed that both candy samples were free of heavy metal contaminants such as Pb, Ti, Hg, Ni, As, Si and Mo. However, Cu was detected in the plantain-based candy sample since plantain is reported to be a source of this micronutrient (*50*).

The results of the microbiological and EDX analyses assured that the formulated candy was completely safe for human consumption and could therefore be subjected to sensory evaluation.

### Sensory and physicochemical characteristics of control sample and plantain-based candies

Consumer acceptance is the most important criterion for a new food product. The hedonic scores from the panel responses for the control sample and the plantain-based candies are shown as radar plots (Fig. 1c and Fig. 1d, respectively). It was found that the panellists accepted well the plantain-based candies due to their uniform dark brown colour (L*=48.5, a*=5.1, b*=11.6, c*=12.7, *h*=66.5), rich mouthfeel, pleasant aroma, moderately hard texture and moderate sweetness. The typical characteristic flavour of green plantains in the processed candy was also moderately appreciated. The control sample mimicked the dark caramel-like colour (L*=51, a*=3.9, b*=10.8, c*=11.5, *h*=70.1) (lighter than plantain-based candies) and texture of boiled candy, tasted extremely sweet and was sticky in the mouth, while plantain-based candies were less hard (more brittle) and not sticky. The caramel-like appearance of the newly developed candies was consistent with the findings of Ronda *et al.* (*51*), who reported that the use of polyols (sorbitol and mannitol) in cake preparation darkened the crust compared to the cakes prepared with sucrose, mainly due to the classic Maillard reaction. In addition, the presence of brown-coloured lyophilized plantain powder also contributed to the formation of a darker colour of the plantain-based candies than of the control sample. The results of TPA (plantain-based candies had hardness ((6804±391) g), adhesiveness ((−238.8±15.8) g·s), springiness (0.14±0.01), cohesiveness (0.41±0.02), gumminess (196.5±32.9) and chewiness (95.8±2.3)) were in good agreement with the sensory attributes. The control sample showed significantly (p<0.05) higher hardness ((10643±893) g), adhesiveness ((−3034±147) g·s), springiness (0.97±0.03), cohesiveness (0.18±0.01), gumminess (1899±156) and chewiness (1848±124) than the plantain-based candies. However, another sugar-free nutraceutical boiled candy made from isomalt, maltitol syrup, xylitol and extract of melon berry (*20*) had a harder texture than the candy developed in this study. Therefore, the newly formulated designer candy was labelled as ‘semi-hard’.

The FE-SEM image of the control sample (Fig. 1e) showed a continuous, uniform, compact and less porous structure with a smooth surface, which corresponded well with the textural properties of the sugar-based hard candies (*52*). In contrast, plantain-based candies (Fig. 1f) showed a grainy, non-homogeneous and discontinuous (with voids) microstructure due to the complex composition of plantains (especially the soluble and insoluble fibre content). There is a lack of similar data on sugar-free hard or semi-hard candies to compare these results.

Fig. 1g and Fig. 1h show the XRD graphs of the control sample and the plantain-based candies, respectively. Both graphs show some nanocrystalline structures on a predominantly amorphous base. The candies were therefore inherently amorphous by nature since they were sugar- and fat-free candies and free of classical defects such as graininess and crystals. However, they were opaque and slightly granular due to the presence of insoluble plantain components (although the same were not the classical ‘blooms’). The control sample was thus categorised as ‘grainy hard candy’ (*24*) and the plantain-based candy as a ‘grainy semi-hard candy’, the latter being relatively more brittle than the former. These data could not be compared or validated with literature reports as, to the best of our knowledge, there are no existing XRD data for sugar-free hard candy.

### Thermal stability of control sample and plantain-based candies

The TGA graphs of both the control sample and the plantain-based candies could be classified as ‘multi-stage decomposition’ type (3-step decomposition), with the first decomposition observed at 125 °C for both candies (Fig. 1i and Fig. 1j), indicating that both candies were thermostable up to very high temperatures.

The DSC thermograms (Fig. 1k) show that the onset of glass transition temperature (*T*_g_) of the control sample was −50 °C, the *T*_g_ midpoint was −38 °C and the *T*_g_ endpoint was −28 °C during both the first and second heating phases. On the other hand, *T*_g_ onset, midpoint and endpoint for the plantain-based candies (Fig. 1l) were −35, −30 and −25 °C, respectively, during the first heating phase, and −30, −25 and −20 °C, respectively, during the second heating phase. The higher *T*_g_ value (mid-point) of the control sample confirmed the hard and brittle texture of the candy during mastication, while the lower *T*_g_ value of the plantain-based candy was consistent with the mouthfeel (as assessed by the sensory panel) of the hard brittle candy, which gradually softened with mastication. The melting peak of the plantain-based candy was observed in a relatively wide range of 94–105 °C, indicating slow melting in the mouth, while the melting peak of the control sample was in a narrower range of 90–94 °C during the first heating phase. During the second heating, the control sample showed no change in *T*_g_ values. However, there was a slight shift in the *T*_g_ and heat flow (W/g) curve for the plantain-based candy, possibly due to the presence of polysaccharides, dietary fibre and other complex compounds.

The absence of peaks indicated the lack of crystallinity of the candies (*36*), which was in good agreement with the results of the XRD data. This was due to the replacement of sucrose as the doctoring agent (*24*) by sugar alcohols, resulting in the absence of sugar blooms in the new designer candies. These results of the TGA, DSC analysis and sensory evaluation were similar to those of Wang (*36*), who had conducted extensive research on boiled sugar candies.

### The *a*_w_ value and proximate composition of control sample and plantain-based candies

Although the control was sugar-free, it had an *a*_w_ value of 0.43, which was similar to that of conventional hard sugar candies (0.25-0.40). The *a*_w_ value of the plantain-based candy was 0.57, which was similar to that of soft candies (0.46-0.60) (*53*). These results were in good agreement with the texture analysis data on hardness, based on which the plantain-based candy was categorised as ‘semi-hard’. The plantain-based candy had a significantly higher (p<0.05) *a*_w_ value (Table 2) than the control sample, as the lyophilized plantain powder (not packaged) was hygroscopic (*54*). The higher *a*_w_ value of the plantain-based candies would pose a challenge for shelf life extension, which could be successfully averted by appropriate packaging of the candies (discussed later).

**Table 2:** Proximate composition on fresh mass basis and water activity of control candy sample and plantain candy sample

Analysis parameter	*w*(parameter)/%	
Control candy sample	Plantain candy sample
Moisture	(3.6±0.8)^a^	(14.7±1.5)^b^
Fat	(0.66±0.03)^a^	(0.16±0.06)^b^
Protein	(0.29±0.02)^a^	(2.6±0.4)^b^
Crude fibre	0	(2.2±0.7)^a^
Ash	(0.90±0.02)^a^	(4.2±0.9)^b^
Carbohydrate (by difference)	(94.5±1.4)^a^	(76.2±2.8)^b^
Water activity	(0.43±0.01)^a^	(0.57±0.02)^b^

Data are mean value±S.D., *N*=3. Different letters in a row indicate significant differences at p<0.05 level

The proximate analyses of the control sample and the plantain-based candies (Table 2) showed that the plantain candies had significantly higher mass fractions of moisture (p<0.001), ash (p<0.001), crude fibre (p<0.001) and protein (p<0.001) and significantly lower mass fractions of fat (p<0.001) and total carbohydrate (p<0.001), giving the plantain-based candies higher nutritional value than the control sample.

### Values of TTA, pH and soluble and insoluble solids of control sample and plantain-based candies

The control sample had an alkaline pH=of 8.1, while the plantain-based candy was acidic (pH=4.3). A significantly higher (p<0.05) percentage of TTA expressed as malic acid (0.12 % in the plantain candy sample than 0.09 and in the control sample) and a lower pH (p<0.05) were found for the plantain candy, which could be beneficial for extending shelf life. These results are consistent with the findings documented by Supriyanto *et al.* (*55*), who found the pH of sucrose-free (with xylitol and glucose syrup) hard candies prepared with Javanese long pepper extract to be 4.3. The control sample consisted mostly of soluble solids (82 %), while the crude fibre content of the plantains (mostly from their peels) contributed to a significantly higher (p<0.05) mass fraction of insoluble solids (36.26 %) in the plantain candies. The higher mass fractions of insoluble solids and crude fibre in the plantain-based candies justified the higher mass of the plantain-based candy, although it was similar in size to the control sample.

### Antioxidant properties of plantain-based candy

The plantain-based candies had a considerable amount of reducing power expressed as butylated hydroxytoluene (BHT) on dry mass (dm) basis (11.30 µg/g), TPC expressed as gallic acid equivalents (GAE) on dm (679 mg/100 g), DPPH radical scavenging activity expressed as IC_50_ value (6.23 mg/mL) and FRAP expressed as FeSO_4_ on dm (2566 mM/100 g), although the processing loss was 18.5, 11.6 and 25.4 %, respectively, compared to the values obtained for raw plantains. Compared to the values obtained for the lyophilized plantain powder, they were 21.51, 1012.5 and 53.59 %, respectively. The TPC value, DPPH radical scavenging activity and FRAP values were significantly (p<0.05) lower in the plantain-based candies compared to the raw lyophilized plantains. These results are in good agreement with those of Dadwal *et al.* (*56*), who found a significant decrease (p<0.05) in TPC, DPPH and FRAP values in candies prepared from bamboo shoots compared to the corresponding values of fresh bamboo shoots. These differences could be due to the treatment/processing that the ingredients underwent during candy production. Since phenolic content is closely related to antioxidant activity, the reduction in TPC during candy production also led to a reduction in DPPH radical scavenging activity and FRAP values of plantain-based candies (*56*).

The contents of l-tryptophan, serotonin and melatonin in the plantain-based candies were 4.54, 1.83 and 1.23 µg/g, respectively, with a synergistic effect value >1, suggesting that the natural synergism among these three biomolecules is maintained in the processed candies. This result is in agreement with the findings of Chakraborty and Bhattacharjee (*21*), who reported on the preservation of antioxidant synergism in processed food products in a nutraceutical beverage formulated with an ultrasound-assisted solvent extract of mustard seeds with lemon and citric acid.

### Best packaging laminate for the extension of the shelf-lives of control sample and plantain-based candies

During storage, the growth of microbes in the candies packaged in the two laminates was restricted for the first three weeks. However, on day 28 of storage, both types of candies (control and plantain-based) packed in NHS BOPP/MET BOPP had considerable bacterial and yeast/mould counts (data not shown), which were above the safe limit for consumption as per FSSAI guidelines (*49*). The candies packaged in NHS BOPP/MET BOPP were therefore discarded on day 28. This study confirmed the suitability of PET/MET PET/PE as a packaging laminate for the control sample and the plantain-based candies.

Based on the properties of the foil wrapper (Table 1), it was found that the values of the mechanical properties (thickness, grammage and tensile strength) for the PET/MET PET/PE laminate (Fig. S1) were significantly higher (p<0.001) and the OTR value was significantly lower (p<0.001) than for the NHS BOPP/MET BOPP laminate (Fig. S1). The high values for thickness, grammage and tensile strength of the three-ply PET/MET PET/PE laminate for flexible packaging indicate improved mechanical strength, while a low OTR value indicates a good barrier property against oxygen and thus a possible prevention of oxidative degradation of the antioxidants contained in the candy. An insignificant difference between the WVTR values indicates a similar permeability of the packaging films to atmospheric water vapour from the atmosphere. The low OTR and WVTR values of the PET/MET PET/PE laminates helped to prevent moisture loss in the candies and thus prevented them from becoming soft (and thus sticky). These properties of the wrapper foils proved to be beneficial for maintaining the organoleptic wholesomeness of the candies and made them safe in terms of microbial bioburden.

The control candies packaged in PET/MET PET/PE were microbiologically safe for up to 56 days, while the plantain-based candies were safe for consumption for up to 63 days (Table S2). Based on these findings, the storage study period for the candy packaged in the best packaging laminate was ascertained to be 63 days post-packaging in PET/MET PET/PE laminate. A similar packaging laminate (PET/MET PET/PE) is reportedly used for commercial packaging of powdered spice mixes (for export) imparting it a shelf life of 9-12 months under normal storage conditions (*57*).

### Shelf life of the candies wrapped in 3-ply PET/MET PET/PE

#### Alterations in sensory attributes during storage

**Fig. 2:**
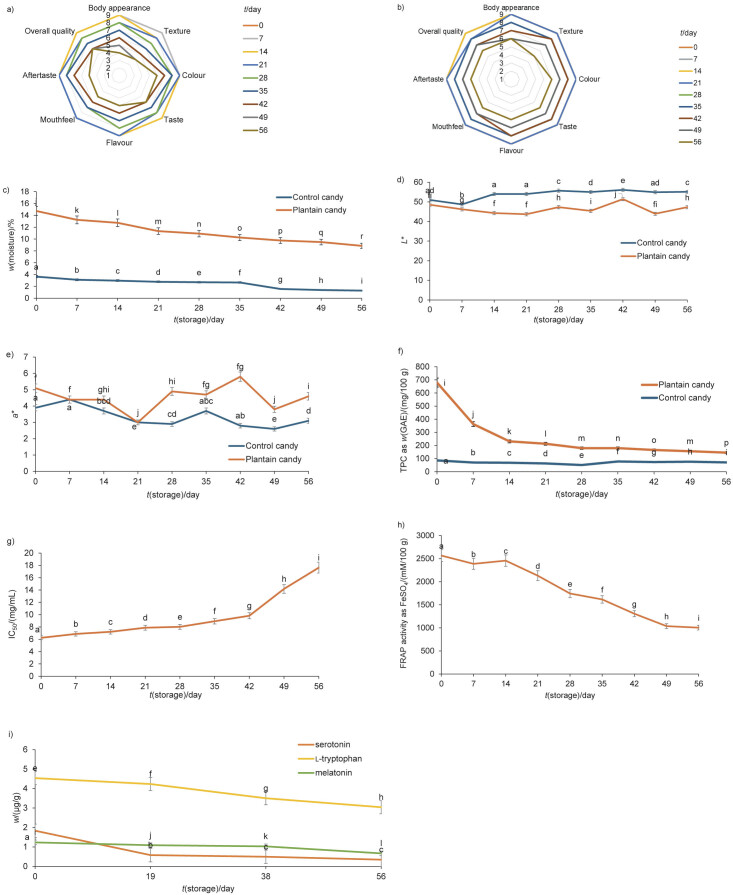
Radar plots of hedonic scores obtained by sensory analyses of: a) control candy sample and b) plantain candy sample during storage at temperature=(27±2) °C and RH=(80±2) %, c) alterations in moisture mass fraction (on wet mass basis) during storage of control candy sample and plantain candy sample. Alterations in colour parameters: d) L* value and e) a* value of control candy sample and plantain candy sample during storage. Phytochemical analysis of plantain candy sample during storage on dry mass basis: f) TPC=total phenolic content, g) IC_50_ value by DPPH, h) FRAP=Fe(III) reducing antioxidant power value, i) l-tryptophan, serotonin and melatonin mass fractions. For high-performance liquid chromatography analyses results RSD≤3 %. The limits of quantification for l-tryptophan, serotonin and melatonin were 1.88, 0.89, and 0.51 μg/L, respectively. Each value is mean value±S.D., *N*=3. Values marked with different letters in superscript are significantly different at p<0.05

The response scores of the sensory properties of the control sample and the plantain-based candies during storage are shown as radar plots in Fig. 2a and Fig. 2b, respectively. On day 56, the plantain-based candy was disapprovingly dry and brittle to the sensory panel, and the control sample was also sensorially unacceptable, due to its increased stickiness and hardness on day 49 of storage. A reduction in the sensory properties of hard candies containing *Basella alba* (Malabar spinach) extract was reported when they were wrapped in a 2-ply laminate of non-stick paper or in aluminium foil and stored in airtight containers at ambient conditions (*58*). The authors attributed the decreases in the sensory scores to deteriorating changes in hardness and adhesiveness, which is consistent with the results of this study. Therefore, in this study, the shelf life of plantain-based candies packaged in PET/MET PET/PE was set at 56 days, although they were microbiologically safe up to 63 days.

#### Changes in physicochemical properties during storage

A significant (p<0.001) decrease in mass fraction of moisture was observed in the plantain-based candies and the control sample during storage (Fig. 2c) from day 0 to the end of the respective shelf life, possibly due to the migration of moisture through the wrapper(s) into the immediate environment (*53*). Moreover, significant differences (p<0.001) were also observed between the mass fraction of moisture in the control sample and the plantain-based candies on each evaluation day. Significant (p<0.001) decreases in texture parameters (such as hardness, adhesiveness, springiness, cohesiveness, gumminess and chewiness) occurred throughout the storage period in both the control sample and the plantain-based candies, with greater changes in the plantain-based candies than in the control (Table 3). Very small changes (p<0.001) were observed in the L*, a* and b* values (Fig. 2d and Fig. 2e) for both the control and plantain candies during storage.

**Table 3:** Texture profile of control candy sample and plantain-based candy sample during storage

*t*/day	Hardness/g	Adhesiveness/(g·s)	Springiness	Cohesiveness	Gumminess	Chewiness
Control candy sample						
0	(10643±893)^a^	(-3034±147)^a^	(0.97±0.03)^a^	(0.18±0.01)^a^	(1899±156)^a^	(1848±124)^a^
7	(10653±683)^b^	(-3075±285)^b^	(0.99±0.03)^b^	(0.19±0.02)^b^	(1893±135)^a^	(1840±131)^b^
14	(10514±844)^c^	(-3012±216)^c^	(0.97±0.05)^c^	(0.18±0.01)^c^	(1887±128)^b^	(1824±103)^c^
21	(10541±580)^d^	(-2987±194)^d^	(0.95±0.02)^d^	(0.17±0.02)^c^	(1872±117)^b^	(1798±116)^d^
28	(10442±719)^e^	(-2964±201)^e^	(0.92±0.01)^e^	(0.17±0.02)^d^	(1856±123)^c^	(1782±119)^e^
35	(10476±904)^f^	(-2993±178)^f^	(0.93±0.06)^d^	(0.17±0.02)^df^	(1839±169)^d^	(1777±136)^f^
42	(10344±729)^g^	(-2955±255)^g^	(0.91±0.06)^f^	(0.16±0.01)^c^	(1823±105)^d^	(1763±148)^g^
49	(10243±812)^h^	(-2913±242)^h^	(0.90±0.08)^g^	(0.19±0.02)^c^	(1816±122)^c^	(1758±152)^h^
56	(10181±914)^i^	(-2879±268)^d^	(0.90±0.03)^g^	(0.16±0.02)^f^	(1804±108)^d^	(1753±150)^h^
Plantain candy sample						
0	(6804±391)^j^	(-238.8±15.8)^i^	(0.14±0.01)^h^	(0.41±0.02)^g^	(196.5±32.9)^e^	(95.8±2.3)^i^
7	(6889±268)^k^	(-242.3±17.2)^j^	(0.18±0.02)^i^	(0.07±0.03)^h^	(486±26)^e^	(85.2±4.2)^j^
14	(6743±342)^l^	(-175.6±10.5)^k^	(0.16±0.02)^j^	(0.06±0.01)^i^	(370±21)^f^	(64.2±1.5)^k^
21	(6535.92±184)^m^	(-115.1±9.9)^l^	(0.11±0.01)^k^	(0.06±0.01)^i^	(416±21)^f^	(46.22±275)^l^
28	(6316±165)^n^	(-125.9±11.1)^m^	(0.15±0.01)^l^	(0.04±0.01)^j^	(228±19)^g^	(33.3±2.1)^m^
35	(6440±228)^o^	(-130.1±4.9)^n^	(0.13±0.01)^k^	(0.05±0.01)^jk^	(360.1±2.1)^h^	(45.3±3.2)^n^
42	(6325±271)^p^	(-103.0±7.2)^o^	(0.09±0.02)^m^	(0.07±0.01)^i^	(234±17)^h^	(21.4±1.3)^o^
49	(6058±193)^q^	(-120.1±9.9)^p^	(0.11±0.01)^n^	(0.05±0.01)^i^	(315±28)^g^	(37.3±3.0)^p^
56	(4683±158)^r^	(-223.5±5.3)^q^	(0.12±0.01)^n^	(0.07±0.01)^k^	(340±23)^h^	(40.3±3.1)^p^

Data are mean value±S.D, *N*=3. Different letters in a column indicate significant differences at p<0.05 level

The decrease in moisture mass fraction during the storage of plantain-based candies explains the appearance of dryness, brittleness and incoherence in the structure of the candy on the last day of storage. Candies tend to lose moisture to the environment as the moisture migrates out of the candy and through the package, making them harder (*53*, *58*). In this study, the plantain-based candy became brittle instead of harder, probably due to the presence of voids inside, as confirmed by analysing its microstructure (Fig. 1f). The loss of moisture also led to a loss of adhesiveness (*53*) and thus to losses in springiness, cohesiveness, gumminess and chewiness. The changes in colour parameters were possibly due to the non-enzymatic browning (*58*) and may have been accelerated by arabinose, an abundant glycoside in one of the constituents of candy, *i.e.* gum acacia (*59*). The results of the present study are consistent with the previously described results of Yan *et al.* (*58*).

#### Changes in antioxidant properties and synergistic effects of antioxidants (l-tryptophan, serotonin and melatonin) during storage

The plantain candy showed a considerable TPC, IC_50_ value of DPPH radical scavenging activity, and FRAP value on day zero, which decreased significantly with storage (Fig. 2f, Fig. 2g and Fig. 2h) until the end of the shelf life (56 days). A similar trend of decrease in TPC and antioxidant activity during storage was reported by Šeremet *et al.* (*60*) in candies containing steviol glycosides, sorbitol, inulin, psyllium, citric acid and white tea extract. At the molecular level, l-tryptophan, serotonin and melatonin contents decreased significantly (p<0.001) from their respective mass fractions (4.54, 1.83 and 1.23 µg/g) on day 0 to the last day of shelf life (3.04, 0.21 and 0.67 µg/g) of the plantain candies (Fig. 2i). The antioxidant effect of the plantain candies was greater than of the individual antioxidants, which means that the natural antioxidant synergy was not disturbed during the processing of candies and also remained unaffected by the physicochemical changes in the candies during storage (Table 4). Thus, the plantain candy would be a rich source of the antioxidant triad – l-tryptophan, serotonin and melatonin – even after 56 days of storage in a commercial foil wrapper. We believe that the processing and packaging of candies presented in this paper represents a sustainable solution for the delivery of plantain-based antioxidant sweets.

**Table 4:** The *in vitro* synergistic effect of serotonin (S), l-tryptophan (T) and melatonin (M) of plantain candy sample

Sample	Serotonin	*γ*/(μg/mL) l-tryptophan	Melatonin	Synergistic effect
S1	1.87	0.00	0.00	-
S2	0.35	0.00	0.00	-
T1	0.00	4.54	0.00	-
T2	0.00	3.04	0.00	-
M1	0.00	0.00	1.23	-
M2	0.00	0.00	0.67	-
Plantain candy sample (*t*=0)	1.87	4.54	1.23	(1.03±0.05)^a^
Plantain candy sample (*t*=56 day)	0.35	3.04	0.67	(1.01±0.07)^b^

Data are mean value±S.D., *N*=3. Different letters in a column indicate significant differences at p<0.05 level

## CONCLUSIONS

The newly developed plantain-based candy could be a promising nutraceutical confectionery rich in three therapeutically important antioxidants, namely l-tryptophan, serotonin and melatonin, which can be safely stored for 56 days without physicochemical and considerable antioxidant deterioration when packaged in a 3-ply flexible (PET/MET PET/PE) laminate wrapper. We believe that this candy can be a vehicle for *in vivo* delivery of these biomolecules as the formulation contains a sugar-free base (of sorbitol and mannitol) and minimal food additives other than a thickener, a desiccant and an anti-fungal agent) with considerable shelf life. This semi-hard candy could be a novel, potentially antioxidant-rich food supplement, especially for the geriatric population.

## Appendix

**Table S1:** Details of ingredients used in candy formulation

Ingredient	Function	Permitted amount	Used amount	Reference
Sorbitol	Low calorie sweetening agent, forms the body of candy	GMP (FSSAI)	*m*(sorbitol):*m*(d-mannitol)=3:1	(*27*)
d-mannitol	Low calorie sweetening agent, forms the base of candy	GMP (FSSAI)		
Gum acacia	Thickening agent, stabilizer, emulsifier	-	30 %	(*26*)
SiO_2_	Desiccant, anticaking agent	-	0.5 %	(*26*)
Sorbic acid	Antifungal agent	1 g/L (FSSAI)	0.1 %	(*26*)
Lyophilized green plantain powder	Principle fortifying material rich in antioxidants	-	65 %	-
Potable water	To solubilize the ingredients	-	50 %	-
Vanilla essence	Flavouring agent	-	100 μL	-

FSSAI=Food Safety and Standards Authority of India, GMP=good manufacturing practices

**Table S2:** Microbial counts in PET/MET PET/PE packaged candies during storage

	*N*/(CFU/g)			
*t*/day	Control candy sample		Plantain candy sample	
	Bacteria	Yeast/mould	Bacteria	Yeast/mould
0	0^a^	0^k^	0^a^	0^k^
7	0^a^	0^k^	0^a^	0^k^
14	0^a^	0^k^	0^a^	0^k^
21	0^a^	0^k^	0^a^	0^k^
28	(187.5±30.0)^b^	0^k^	0^a^	0^k^
35	(250±78)^c^	0^k^	(187.5±36.0)^b^	0^k^
42	(500±84)^d^	0^k^	(437.5±73.0)^g^	0^k^
49	(562.5±96.0)^e^	0^k^	(812.5±94.0)^h^	0^k^
56	(1750±187.0)^f^	(937.5±80.0)^l^	(687.5±69.0)^i^	0^k^
63	-	-	(1062.5±122.0)^j^	(312.5±116.0)^m^

Data are mean value±S.D., *N*=3. Different letters indicate significant differences at p<0.05 level. PET=polyethylene terephthalate; MET PET=metallized polyester; PE=polyethylene

## References

[1] Oyeyinka BO Afolayan AJ. Comparative evaluation of the nutritive, mineral, and antinutritive composition of *Musa sinensis* L. (banana) and *Musa paradisiaca* L. (plantain) fruit compartments. Plants. 2019;8(12):598. 10.3390/plants8120598 31842474 PMC6963461

[2] Sarkar P Tamili D Bhattacharjee P. Low dose gamma‐irradiation enhances shelf‐life and contents of serotonin and melatonin in green plantains (*Musa paradisiaca*): A study involving antioxidant synergy. J Food Process Preserv. 2021;45(11):e15934. 10.1111/jfpp.15934

[3] Kumar PS Uma S. Banana processing: A silver lining during corona commotion. Biot Res Today. 2020;2(5):305–7.

[4] Babayemi JO Dauda KT Kayode AA Nwude DO Ajiboye JA Essien ER Determination of potash alkali and metal contents of ashes obtained from peels of some varieties of Nigeria grown *Musa* species. BioResources. 2010;5(3):1384–92. 10.15376/biores.5.3.1384-1392

[5] Akubor PI Adamolekun FO Oba CA Obari H Abudu IO. Chemical composition and functional properties of cowpea and plantain flour blends for cookie production. Plant Foods Hum Nutr. 2003;58:1–9. 10.1023/B:QUAL.0000041160.25384.f612859008

[6] Sarawong C Gutiérrez ZR Berghofer E Schoenlechner R. Effect of green plantain flour addition to gluten‐free bread on functional bread properties and resistant starch content. Int J Food Sci Technol. 2014;49(8):1825–33. 10.1111/ijfs.12491

[7] Onwuka GI Onwuka ND. The effects of ripening on the functional properties of plantain and plantain based cake. Int J Food Prop. 2005;8(2):347–53. 10.1081/JFP-200059489

[8] Duyff RL Birch LL Byrd-Bredbenner C Johnson SL Mattes RD Murphy MM Candy consumption patterns, effects on health, and behavioral strategies to promote moderation: summary report of a roundtable discussion. Adv Nutr. 2015;6(1):139S–46S. 10.3945/an.114.007302 25593156 PMC4288276

[9] Other ingredients. In: Alikonis JJ, editor. Candy technology. West Port, Connecticut, USA: The AVI Publishing Company Inc; 1979. pp. 42-3.

[10] Gámez-Meza N Noriega-Rodríguez JA Medina-Juárez LA Ortega-García J Cázarez-Casanova R Angulo-Guerrero O. Antioxidant activity in soybean oil of extracts from Thompson grape bagasse. J Am Oil Chem Soc. 1999;76(12):1445–7. 10.1007/s11746-999-0182-4

[11] Souiy Z Amri Z Sharif H Souiy A Cheraief I Hamden K The use of D-optimal mixture design in optimizing formulation of a nutraceutical hard candy. Int J Food Sci. 2023;2023: 10.1155/2023/7510452 36968159 PMC10033211

[12] Mahato A Chakraborty I Baidya BK. Preparation and evaluation of fruit candy from unripe mango. Int J Chem Stud. 2020;8(1):2727–31. 10.22271/chemi.2020.v8.i1ao.8682

[13] Bartkiene E Zokaityte E Zavistanaviciute P Mockus E Cernauskas D Ruzauskas M Nutraceutical chewing candy formulations based on acetic, alcoholic, and lactofermented apple juice products. Foods. 2021;10(10):2329. 10.3390/foods10102329 34681378 PMC8535157

[14] Bartkiene E Tolpeznikaite E Klupsaite D Starkute V Bartkevics V Skrastina A Bio-converted *Spirulina* for nutraceutical chewing candy formulations rich in l-glutamic and gamma-aminobutyric acids. Microorganisms. 2023;11(2):441. 10.3390/microorganisms11020441 36838408 PMC9959499

[15] Cano-Lamadrid M Calín-Sánchez Á Clemente-Villalba J Hernández F Carbonell-Barrachina ÁA Sendra E Quality parameters and consumer acceptance of jelly candies based on pomegranate juice “Mollar de Elche”. Foods. 2020;9(4):516. 10.3390/foods9040516 32325998 PMC7230151

[16] Delgado P Bañón S. Effects of replacing starch by inulin on the physicochemical, texture and sensory characteristics of gummy jellies. CYTA J Food. 2018;16(1):1–10. 10.1080/19476337.2017.1327462

[17] Cedeño-Pinos C Martínez-Tomé M Murcia MA Jordán MJ Bañón S. Assessment of rosemary (*Rosmarinus officinalis* L.) extract as antioxidant in jelly candies made with fructan fibres and stevia. Antioxidants. 2020;9(12):1289. 10.3390/antiox9121289 33339389 PMC7767232

[18] Cedeño-Pinos C Marcucci MC Bañón S. Contribution of green propolis to the antioxidant, physical, and sensory properties of fruity jelly candies made with sugars or fructans. Foods. 2021;10(11):2586. 10.3390/foods10112586 34828866 PMC8620292

[19] Cedeño-Pinos C Martínez-Tomé M Jordán MJ Bañón S. Revalorisation of sage (*Salvia lavandulifolia* Vahl) by-product extracts as a source of polyphenol antioxidants for novel jelly candies. Antioxidants. 2023;12(1):159. 10.3390/antiox12010159 36671021 PMC9854814

[20] Jeon Y Oh J Cho MS. Formulation optimization of sucrose-free hard candy fortified with *Cudrania tricuspidata* extract. Foods. 2021;10(10):2464. 10.3390/foods10102464 34681513 PMC8536104

[21] Chakraborty S Bhattacharjee P. Design of lemon–mustard nutraceutical beverages based on synergism among antioxidants and *in vitro* antioxidative, hypoglycemic and hypocholesterolemic activities: Characterization and shelf life studies. J Food Meas Charact. 2018;12:2110–20. 10.1007/s11694-018-9826-0

[22] Messina M Lampe JW Birt DF Appel LJ Pivonka E Berry B Reductionism and the narrowing nutrition perspective: time for reevaluation and emphasis on food synergy. J Am Diet Assoc. 2001;101(12):1416–9. 10.1016/S0002-8223(01)00342-X 11762736

[23] Bhattacharya A. Ultra-processed foods may cause mental health decline, says study. The Times of India, Kolkata, Bennett, Coleman & Co. Ltd. 2023. p. 8.

[24] Confectionary processes and formulations. In: Minifie BW, editor. Chocolate, cocoa and confectionery: Science and technology. New York, USA: Van Nostrand Reinhold; 1989. pp. 530-55.

[25] Dal-Bó V Freire JT. Effects of lyophilization on colorimetric indices, phenolics content, and antioxidant activity of avocado (*Persea americana*) pulp. Food Control. 2022;132:108526. 10.1016/j.foodcont.2021.108526

[26] FSSAI. Appendix A: List of Food additives; 2011. Available from: https://fssai.gov.in/upload/uploadfiles/files/appendix_a_and_b_revised (30-12-2011).pdf.

[27] Roger D, editor. Food technology review No 5. Park Ridge, NJ, USA: Noyes Data Corporation; 1973. pp. 262-5.

[28] Dwivedi BK. Sorbitol and mannitol. In: Nabors LO, Gelardi RC, editors. Alternative sweeteners. New York, NY, USA: Marcel Dekker Inc; 1991. p. 338.

[29] Chéron JB, Axel M, Sébastien F. Natural sweeteners. In: Melton L, Shahidi F, Varelis P, editors. Encyclopedia of food chemistry, vol. 1. Amsterdam, The Netherlands: Elsevier Inc; 2019. pp. 189-95.

[30] Pepper T. Alternative bulk sweeteners. In: Jackson EB, editor. Sugar confectionery manufacture. New York, NY, USA: Van Nostrand Reinhold; 1990. pp. 17-27.

[31] Bose A Tamili D Jana A Bhattacharyya N Bhattacharjee P. l-proline enrichment of bread enhances its KFO: Assessment of freshness by electronic nose technology and an ANN prediction model. Appl Food Res. 2023;3(1):100292. 10.1016/j.afres.2023.100292

[32] ASTM E1871-17Standard guide for serving protocol for sensory evaluation of foods and beverages. West Conshohocken, PA, USA: ASTM International; 2017. 10.1520/E1871-17 10.1520/E1871-17

[33] Ranganna S, editor. Handbook of analysis and quality control for fruit and vegetable products. New Delhi, India: Tata McGraw-Hill Education Private Limited;1986. pp. 9-10.

[34] Stone H, Sidel JL, editors. Sensory evaluation practices. Redwood City, CA, USA: Elsevier Academic Press; 2004. pp. 87-90.

[35] Exponent Lite Express Software, v. 6.1.4.0, Stable Micro Systems Ltd, Godalming, UK; 2013. Available from: https://www.stablemicrosystems.com/SoftwareUpdateExponentLiteExpress.html.

[36] Wang M. Thermal behavior characterization of a sugar-based model system and commercial confections across the stages of sugar cooking [MSc Thesis]. Urbana, IL, USA: University of Illinois; 2017.

[37] Official Method AOAC. 978.18-1978. Water activity of canned vegetables. Rockville, MD, USA: AOAC International; 1978.

[38] Official Method AOAC. 934.01-1934. Loss on drying (moisture) at 95 °C-100 °C for feeds. Rockville, MD, USA: AOAC International; 1998.

[39] Official Method AOAC. 920.87-1920. Protein (total) in flour. Rockville, MD, USA: AOAC International; 1995.

[40] AOAC official Method 920.39-1920. Fat (crude) or ether extract in animal feed. Rockville, MD, USA: AOAC International; 2000.

[41] Method AACC. 08-01.01. Ash – basic method. St. Paul, MN, USA: Cereals & Grains Association; 2000.

[42] AOAC official method 978.10-1979. Fiber (crude) in animal feed and pet food. Fritted glass crucible method. Rockville, MD, USA: AOAC International; 2005.

[43] Bhattacharjee P Das S. 1,8-Cineol-rich cardamom seed (*Elettaria cardamomum*) extracts using green technologies and conventional extractions: Process analysis, phytochemical characterization, and food application. Sep Sci Technol. 2015;50(13):1974–85. 10.1080/01496395.2015.1016038

[44] Aiyegoro OA Okoh AI. Preliminary phytochemical screening and *in vitro* antioxidant activities of the aqueous extract of *Helichrysum longifolium* DC. BMC Complement Altern Med. 2010;10:21. 10.1186/1472-6882-10-21 20470421 PMC2877649

[45] Tamili D Jana S Bhattacharjee P. Chromatographic method development for simultaneous determination of serotonin, melatonin and l-tryptophan: Mass transfer modelling, chromatographic separation factors, and method prediction by artificial neural network. J Chemometr. 2023;37(12):e3520. 10.1002/cem.3520

[46] Peyrat‐Maillard MN Cuvelier ME Berset C. Antioxidant activity of phenolic compounds in 2,2′‐azobis (2‐amidinopropane) dihydrochloride (AAPH)‐induced oxidation: Synergistic and antagonistic effects. J Am Oil Chem Soc. 2003;80(10):1007–12. 10.1007/s11746-003-0812-z

[47] IS 1060-1. Methods of sampling and test for paper and allied products. New Delhi, India: Bureau of Indian Standards; 1966.

[48] IBM SPSS Statistics for Windows, v. 26.0. IBM Corp., Armonk, NY, USA; 2019. Available from: https://www.ibm.com/products/spss-statistics.

[49] Food safety and standards (food products standards and food additives) regulations. New Delhi, India: Food Safety and Standards Authority of India (FSSAI); 2011. Available from: https://www.fssai.gov.in/upload/uploadfiles/files/Compendium_Food_Additives_Regulations_08_09_2020-compressed.pdf.

[50] Okorie DO Eleazu CO Nwosu P. Nutrient and heavy metal composition of plantain (*Musa paradisiaca*) and banana (*Musa paradisiaca*) peels. J Nutr Food Sci. 2015;5(3):1000370. 10.4172/2155-9600.1000370

[51] Ronda F Gómez M Blanco CA Caballero PA. Effects of polyols and nondigestible oligosaccharides on the quality of sugar-free sponge cakes. Food Chem. 2005;90(4):549–55. 10.1016/j.foodchem.2004.05.023

[52] Gu J Ahn‐Jarvis JH Vodovotz Y. Development and characterization of different black raspberry confection matrices designed for delivery of phytochemicals. J Food Sci. 2015;80(3):E610–8. 10.1111/1750-3841.12808 25676542

[53] Ergun R Lietha R Hartel RW. Moisture and shelf life in sugar confections. Crit Rev Food Sci Nutr. 2010;50(2):162–92. 10.1080/10408390802248833 20112158

[54] Naknaen P, Charoenthaikij P, Kerdsup P. Physicochemical properties and nutritional compositions of foamed banana powders (Pisang Awak, *Musa sapientum* L.) dehydrated by various drying methods. Walailak J Sci & Tech. 2016;13(3):177-91. 10.14456/wjst.2016.18 10.14456/wjst.2016.18

[55] Supriyanto MM Ambarwati Y. Supplementation of Javanese long pepper extracts into sucrose-free hard candy for improving antibacterial activity against *Streptococcus mutans.* IOP Conf Ser Earth Environ Sci. 2023;1182:012059. 10.1088/1755-1315/1182/1/012059

[56] Dadwal V Sharma A Joshi R Gupta M. Assessment of nutritional properties and phenolic characterization of freshly harvested *Dendrocalamus hamiltoni* shoots and processed bamboo candy. Food Sci Biotechnol. 2023;32:769–78. 10.1007/s10068-022-01218-5 37041808 PMC10082696

[57] Indiramma AR. Packaging aspects of spices and spice products. In: Plastics in food packaging. Mysore, India: Food Packaging Technology Department, Central Food Technological Research Institute; 2004.

[58] Yan LP Linn NGY Arunasalam M Hui TC Salvamani S Baskaran G. Changes in physicochemical, microbiological and sensory properties of candy incorporated with *Basella alba* upon storage. J Culin Sci Technol. 2022;21(6):981–93. 10.1080/15428052.2022.2026053

[59] do Nascimento RR Pimentel TC Garcia S Prudencio SH. Acacia gum candy with *Limosilactobacillus reuteri* and lemongrass essential oil: Effect of storage time on physicochemical characteristics and probiotic survival. Food Biosci. 2023;56:103128. 10.1016/j.fbio.2023.103128

[60] Šeremet D Mandura A Vojvodić Cebin A Martinić A Galić K Komes D. Challenges in confectionery industry: Development and storage stability of innovative white tea‐based candies. J Food Sci. 2020;85(7):2060–8. 10.1111/1750-3841.15306 32579746

